# 3T cardiac imaging with on-line 12-lead ECG monitoring

**DOI:** 10.1186/1532-429X-18-S1-P212

**Published:** 2016-01-27

**Authors:** Mikayel Dabaghyan, Shelley H Zhang, Jay Ward, Raymond Y Kwong, William G Stevenson, Ronald D Watkins, Zion T Tse, Ehud J Schmidt

**Affiliations:** 1 R&D, Mirtech Inc, Boston, MA USA; 2 grid.62560.370000000403788294Cardiology, Brigham and Women's Hospital, Boston, M USA; 3 E-TROLZ Inc, North Andover, MA USA; 4 grid.168010.e0000000419368956Radiology, Stanford University, Stanford, CA USA; 5 grid.213876.9000000041936738XEngineering, University of Georgia, Athens, GA USA; 6 grid.62560.370000000403788294Radiology, Brigham and Women's Hospital, Boston, MA USA

## Background

We previously demonstrated rapid detection of acute ischemia inside MRI using a prototype MRI-conditional 12-lead ECG system [1] equipped with hardware to remove artifacts produced by the MRI gradients. We also synchronized [2] high-field (1.5-7T) scanners using triggers from 12-lead traces following Magneto-Hydro-Dynamic voltage removal. A commercial 12-lead ECG system might allow performing MR imaging studies with a greater risk of ischemic events, as well as the execution of high-risk MRI-guided interventions, such as ventricular tachycardia ablation.

Our objective is to validate the performance of an alpha-site version of a commercial MRI-compatible 12-lead ECG system during cardiac imaging at 3T.

## Methods

**Figure 1:**
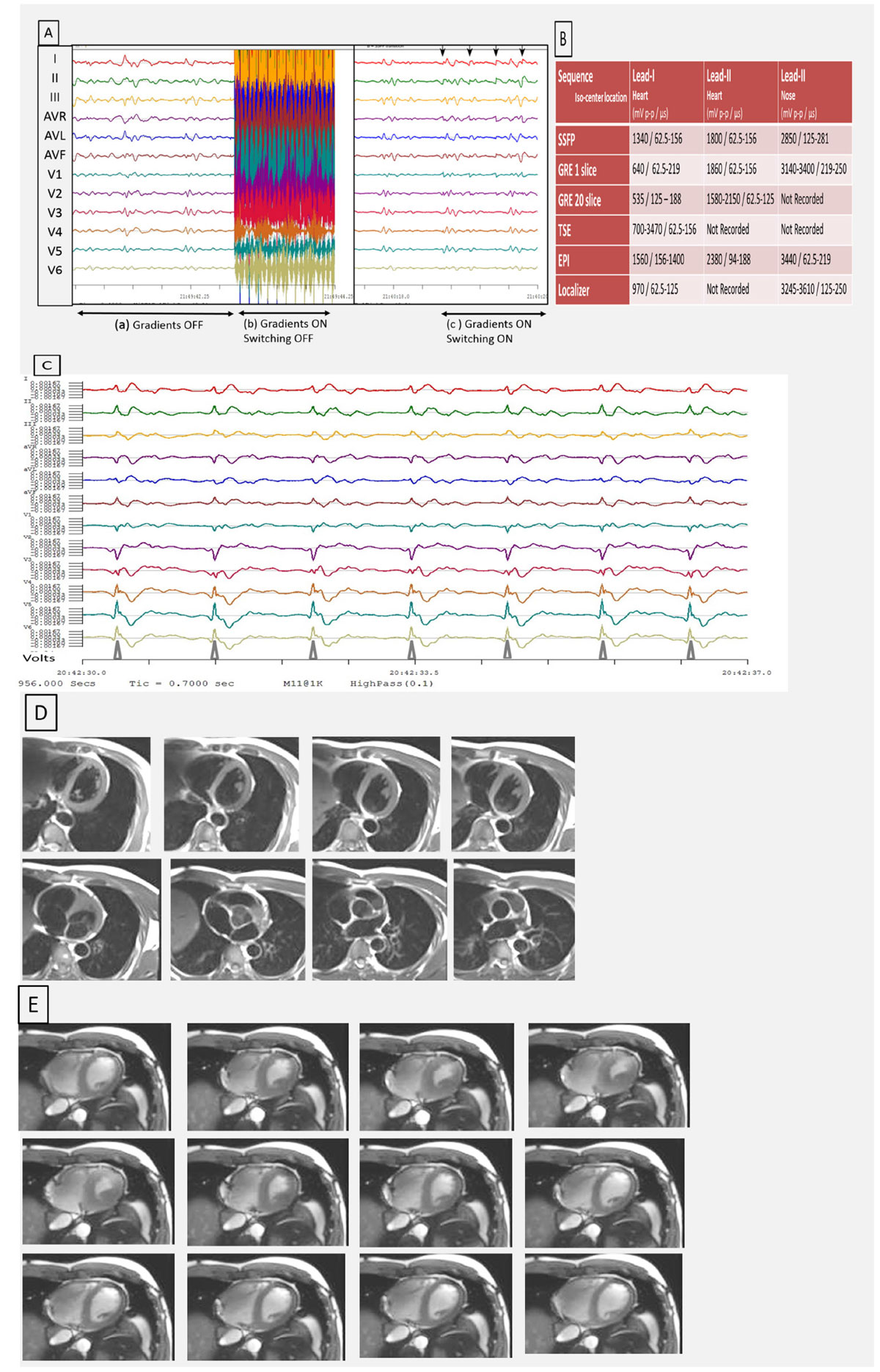
**(A) Artifacts on 12-lead ECG traces during an SSFP sequence with the heart at iso-center**. (a) ECG traces acquired in the MRI without the sequence running, (b) traces acquired during the sequence, which are overlaid with strong gradient-induced voltages, since the hardware switching is disabled, and (c) traces obtained during the sequence, and with the hardware switching enabled, demonstrating the quality of gradient-induced voltage removal. Black arrows indicate regions of largest voltages, which were removed by hardware. Note the similarity of the cleaned ECG traces in (c) with those acquired without imaging in (a). (B) The magnitude of the gradient-induced voltages (sampled at 32 KHz) on the limb leads (channels I-III) can be very large, reaching 1800 mV peak-to-peak (mV p-p) for SSFP with the heart at iso-center (and far larger voltages with peripheral organs, such as the nose, at iso-center). The temporal extent of the largest induced voltages, in microseconds (μs), which is used to tune the sample-and-hold circuits, is listed. These large artifacts are removed by the 12-lead system. (C) Volunteer 12-lead ECG acquired inside the MRI during imaging. Lower row triangles depict location of QRS triggers sent to the MRI scanner’s gating interface. (D) Breath-held volunteer ECG-triggered BB-TSE (TR/TE=2100 ms/46 ms, ETL=9, 192 × 256, 29 × 36, 5 mm sw, 14s/sl., 2 avg.). Acquisition was prospectively triggered by the 12-lead VCG. (E) 12 frames from 25 frame/R-R breath-held volunteer ECG-triggered SSFP cine (TR/TW/flip=3.5 ms/1,2 ms/28deg, 26 × 32, 216 × 256, 5 mm sw, 10s/sl., 2 avg.). Acquisition was retrospectively triggered by the 12-lead VCG.

A pre-release commercial MRI-conditional 12-lead ECG system (MIRTLE, E-TROLZ, Andover, MA) was used in the study of 4 volunteers (32, 35, 42, 57 yrs. old) in a Siemens 3T Skyra during the execution of routine SSFP and GRE cine, as well as Black-Blood TSE cardiac (CMR) imaging. This system removes the largest (>50 milliVolt) ECG gradient-artifacts overlaid on the true 12-lead traces [Figure 1A, B] using hardware switches, together with sample-and-hold (memory) circuits that reduce switching artifacts. The resulting "cleaned" intra-MRI ECG traces are, thereafter, processed by 12-lead vector-cardiogram (VCG) software, which uses ("training") traces acquired outside the MRI to detect the QRS complex in each R-R cycle, despite the presence of Magneto-Hydro-Dynamic voltages on traces inside the MRI. The 12-lead system was used to (a) continuously monitor subjects' ECG inside the bore, including during imaging, and (b) output triggers at each detected QRS complex, which were fed into the scanner's wireless gating system, allowing for acquisition of retrospective and prospective CMR sequences.

We evaluated the fraction of QRS complexes detected (scan efficiency), and the image quality (IQ) obtained using the system [Figure 1B], relative to the scanner's 4-lead gating. We also evaluated the quality of the ECG traces obtained during imaging, relative to ECG traces obtained during imaging pauses [Figure 1C].

## Results

The noise on MRI images was identical (+0.5dB) with the system OFF and ON. Scan efficiency was equivalent (TSE) or ~20% superior (SSFP, GRE) to 4-lead gating. TSE and Cine SSFP IQ was equivalent (Figure 1D, E) to 4-lead-gated.

## Conclusions

The MIRTLE pre-release 12-lead ECG system performed well in imaging and ECG quality tests. The system will now be evaluated in patients at 1.5T and 3T clinical sites.

